# Hepatic Encephalopathy in the Hepato-Gastroenterology and Internal Medicine Department of General Idrissa Pouye Hospital: A Retrospective Prognostic Study of 69 Cases

**DOI:** 10.7759/cureus.92774

**Published:** 2025-09-20

**Authors:** Bibata Toure, Mama Ndiémé Diouf, Mamadou Ngoné Gueye, Nogoye Niang, Abdel Aziz A Fall, Gnagna Diouf, Daouda Dia, Mouhamadou Mbengue

**Affiliations:** 1 Gastroenterology and Hepatology, General Idrissa Pouye Hospital, Dakar, SEN; 2 Gastroenterology and Hepatology, Cheikh Anta Diop University, Dakar, SEN

**Keywords:** acute liver failure, cirrhosis, hepatic encephalopathy, precipitating factors, prognosis, senegal

## Abstract

Introduction: Hepatic encephalopathy (HE) is a common and severe complication of acute or chronic liver failure. The aim of this study was to describe the epidemiological, diagnostic, therapeutic, and outcome aspects of HE in an internal medicine and hepato-gastroenterology department.

Methods: This was a retrospective descriptive study based on data collected over a six-year period (June 2017 to May 2023) at the hepato-gastroenterology and internal medicine department of Idrissa Pouye General Hospital. All patient records with diagnosed HE were collected. Data were recorded using a standardized survey form, entered, and analyzed with IBM SPSS Statistics for Windows, Version 25 (Released 2017; IBM Corp., Armonk, New York, United States). Multivariate analysis was performed using binary logistic regression with a significance level set at p<0.05.

Results: A total of 82 cases of HE were identified among 2480 patients hospitalized during the study period, corresponding to a prevalence of 3.3 %. Thirteen records were unusable, leaving 69 records for analysis. The mean age was 49 ± 16.70 years (range: 17 to 85 years). A male predominance was observed with a sex ratio of 2.45 (49 men, 71.0%). Asterixis (flapping tremor) was present in 41 patients (59.4%). HE was most often classified as West Haven stage II in 41 patients (59.4%). Cirrhosis was present in 57 patients (82.6%), with 51 of these classified as Child-Pugh C (73.9%). The predominant etiology identified among these patients was chronic hepatitis B virus infection. Associated clinical signs were dominated by edematous-ascitic decompensation in 52 patients (75.4%) and jaundice in 51 patients (73.9%). The precipitating factors most frequently associated with the HE episode were herbal medicine use in 22 patients (31.9%), spontaneous bacterial peritonitis (SBP) in 18 patients (26.1%), and gastrointestinal bleeding in 13 patients (18.8%). All patients received treatment based on lactulose and rifaximin. The outcome was marked by death in 53 cases (76.8%), with a mean delay of 7.2 days from symptom onset to death. Multivariate analysis identified a Child-Pugh score of C13 (p=0.043) and the presence of jaundice (p=0.034) as factors associated with poor prognosis.

Conclusion: HE most commonly occurs on a background of severe cirrhosis. Most often precipitated by herbal medicine use, SBP, or gastrointestinal bleeding, the prognosis remains poor, influenced in our study by jaundice and a Child-Pugh C score. Death typically occurs within the first week after symptom onset.

## Introduction

Hepatic encephalopathy (HE) is a severe and potentially fatal complication arising from various liver diseases [1]. It represents a common neuropsychiatric complication associated with hepatocellular insufficiency, particularly in patients with decompensated cirrhosis. HE is characterized as a metabolic brain dysfunction secondary to acute or chronic liver injury, presenting a wide spectrum of cognitive disturbances ranging from mild vigilance impairment to deep coma [2,3].

The primary pathophysiological mechanism involves the accumulation of neurotoxic substances, notably ammonia, which the compromised liver fails to metabolize efficiently. This dysfunction is often exacerbated by precipitating factors such as infections, gastrointestinal hemorrhage, electrolyte imbalances, constipation, and the use of herbal medicines, factors frequently encountered in resource-limited settings [4-6].

Clinically, HE severity is graded using the West Haven classification, which spans from Stage I (characterized by attention deficits and sleep-wake cycle disturbances) to Stage IV (coma) [7]. Diagnosis is primarily clinical, supported, when necessary, by paraclinical investigations including serum ammonia measurements and electroencephalography [8].

HE imposes a significant burden on patients, families, healthcare systems, and society due to its impact on survival, quality of life, and daily functioning [9]. Management hinges on addressing precipitating factors and reducing intestinal ammonia production and absorption, using treatments such as lactulose and non-absorbable antibiotics like rifaximin, alongside supportive care [10]. Nevertheless, prognosis remains poor, especially in cirrhotic patients classified as Child-Pugh class C, who exhibit high mortality rates during severe episodes [11,12].

Reported mortality rates from HE reach 66.6% and 51.6% in Bamako and Madagascar [13,14], respectively, underscoring its public health significance. In Senegal, epidemiological data on HE are scarce. Thus, improved understanding of local characteristics, precipitating factors, management practices, and outcomes is crucial to guiding prevention efforts and optimizing care.

This study aims to describe the epidemiological, diagnostic, therapeutic, and clinical evolution characteristics of HE patients admitted to the Internal Medicine and Hepato-Gastroenterology Department of the Idrissa Pouye General Hospital in Dakar, Senegal.

## Materials and methods

A retrospective descriptive study was conducted over six years (June 2017-May 2023) within the hepato-gastroenterology and internal medicine departments of Idrissa Pouye General Hospital. The study population included all patients aged 18 years and older hospitalized for HE. The diagnosis was established based on an acute onset of altered consciousness ranging from lethargy with apathy, temporal-spatial disorientation, behavioral changes, and asterixis to coma following drug intoxication or in the context of chronic liver disease. Cases with altered consciousness due to other causes were excluded.

Encephalopathies were classified pathophysiologically into those due to acute liver failure and those secondary to chronic liver disease. Clinical severity of HE was assessed using the West Haven classification [2], which grades encephalopathy from Grade 0, indicating no detectable changes in personality or behavior with only minimal changes in memory, concentration, intellectual function, and coordination, to Grade IV, which corresponds to coma unresponsive to verbal or noxious stimuli. Intermediate grades include Grade I with trivial lack of awareness, euphoria or anxiety, shortened attention span, impaired addition or subtraction, and altered sleep rhythm; Grade II characterized by lethargy, apathy, minimal disorientation for time or place, subtle personality change, inappropriate behavior, and asterixis; and Grade III including somnolence to semi-stupor but responsive to stimuli, confusion, and gross disorientation.

The severity of underlying liver disease was evaluated using the Child-Pugh classification [15], which considers five clinical and laboratory parameters: degree of encephalopathy, ascites, serum bilirubin, serum albumin, and prothrombin time or INR. The total score stratifies patients into Class A (5-6 points), representing well-compensated disease; Class B (7-9 points), indicating significant functional compromise; and Class C (10-15 points), denoting decompensated disease.

Data collected included sociodemographic variables (age, sex), clinical and paraclinical findings, encephalopathy grading, precipitating factors, and clinical outcomes. Data were extracted from medical records using standardized forms and entered into a database. Missing data and records with incomplete critical variables were excluded.

Statistical analysis was carried out using IBM SPSS Statistics for Windows, Version 25 (Released 2017; IBM Corp., Armonk, New York, United States). Descriptive statistics summarized patient demographics, clinical characteristics, and outcomes. Bivariable and multivariable analyses employed binary logistic regression with statistical significance defined as p < 0.05.

## Results

A total of 82 cases of HE were identified among 2480 patients hospitalized during the study period, corresponding to a prevalence of 3,3 %. Thirteen records were unusable due to missing data, leaving 69 records for analysis.

**Figure 1:**
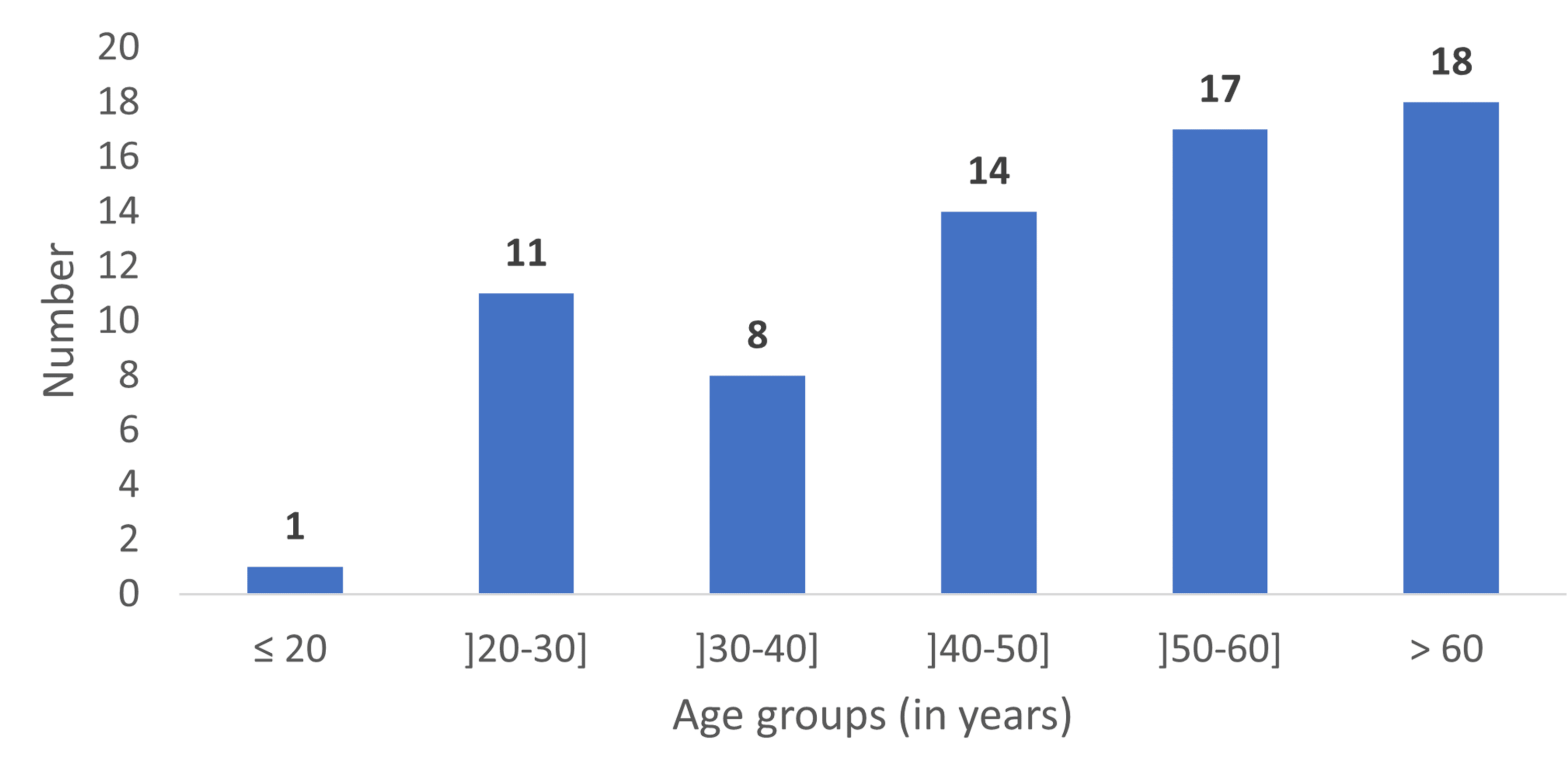
Distribution of patients by age groups (n=69)

The cohort had a mean age of 49 ± 16.70 years, with 71% of individuals aged over 40 years; the largest age group (26.1%) consisted of patients older than 60 years (Figure 1). The sex distribution was characterized by a predominance of male patients, who accounted for 71% (n = 49) of the cohort, resulting in a sex ratio of 2.45.

**Figure 2:**
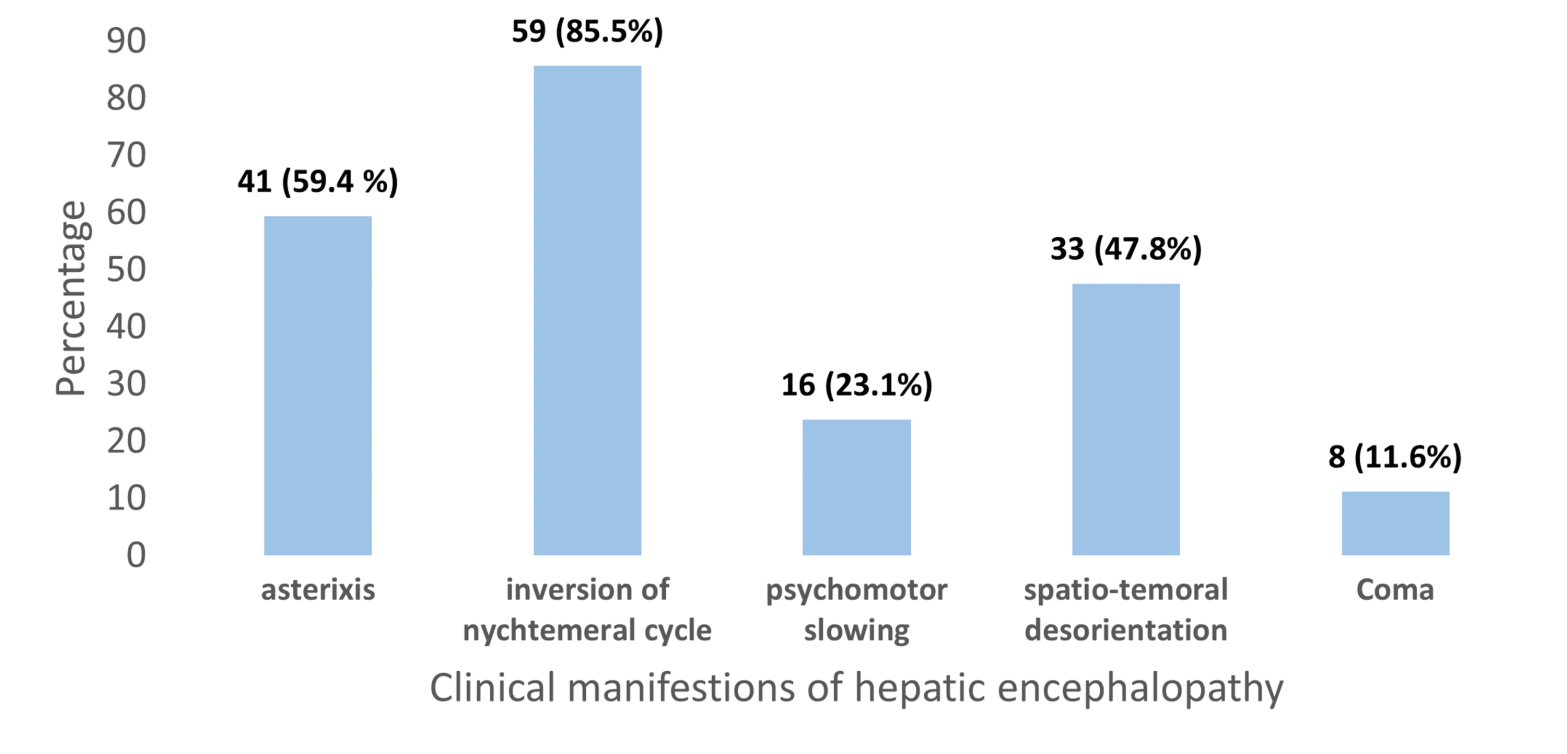
Frequencies of clinical manifestations of hepatic encephalopathy (n= 69 patients)

The most frequently observed clinical manifestation was inversion of nycthemeral rhythm, present in 85.5% of cases. Asterixis was documented in 59.4% of patients. Temporal-spatial disorientation was reported in 47.8%, while psychomotor slowing was found in 23.1% (Figure 2).

**Figure 3:**
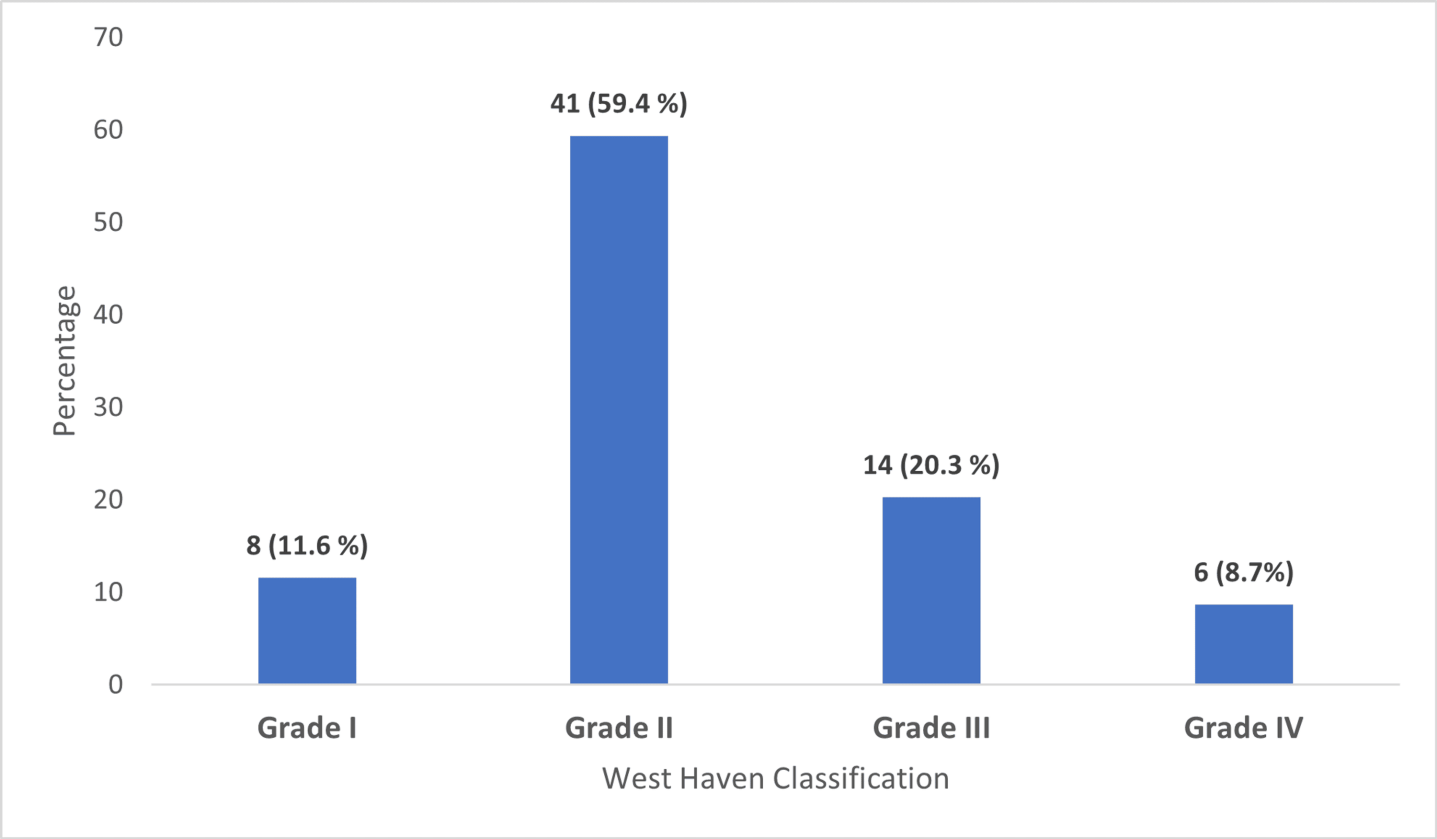
Distribution of patients by West Haven Classification (n= 69 patients)

Most patients were classified as Grade II HE, accounting for 59.4% of cases. Grade III was observed in 20.3% of patients, while Grade I and Grade IV were identified in 11.6% and 8.7% of cases, respectively. Most cases were classified as type C (Figure 3).

Cirrhosis was present in 82.6% of patients, with 8.7% of cirrhotic patients classified as Child-Pugh B and 73.9% as Child-Pugh C. The main etiology among these patients was chronic hepatitis B virus infection in 73,7% of cases. The other etiologies included hepatitis C in one case and autoimmune cirrhosis in one case. The etiology of cirrhosis was not identified in 13 patients. 

Edematous-ascitic decompensation was observed in 75.4%, and jaundice in 73.9% of cases.

**Figure 4:**
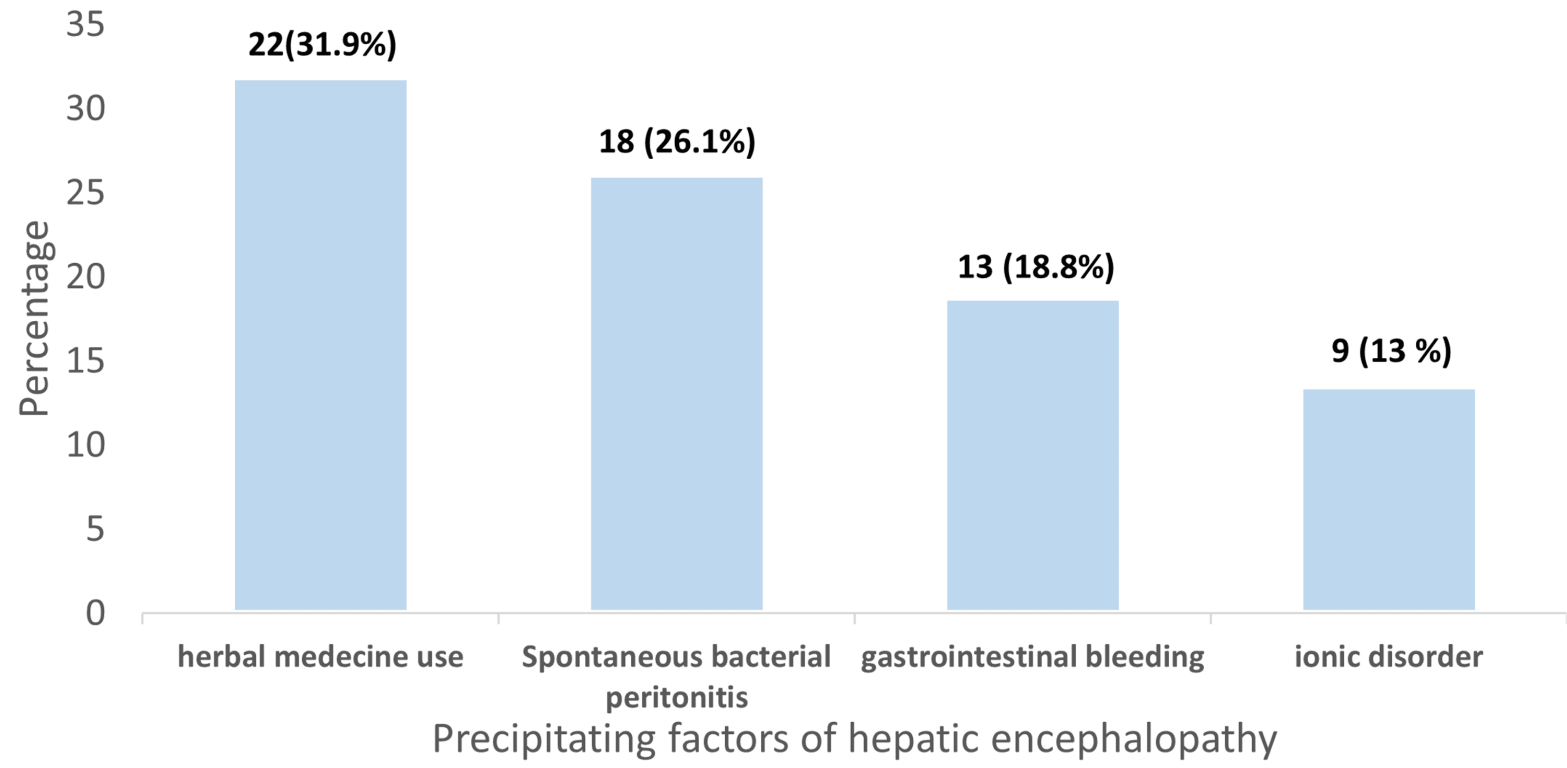
Precipitating factors of hepatic encephalopathy (n=69 patients)

The main precipitating factors identified were herbal medicine use (31.9%), spontaneous bacterial peritonitis (SBP) (26.1%), and gastrointestinal bleeding (18.8%) (Figure 4).

There were eight cases of acute hepatitis, of which six were sub-fulminant and two were fulminant. Toxic and medication-related etiologies accounted for five cases, including two toxic cases, two medication cases, and one hepatitis A virus case, while three cases had no identifiable etiological factor.

All patients received lactulose and rifaximin; additional antibiotic treatment was administered in cases with infection in 53.6 % of cases.

Mortality occurred in 76.8% of patients (n = 53), with a mean time from symptom onset to death of 7.2 days.

Bivariate analysis showed significant associations between mortality and jaundice (OR 6.29), Child-Pugh class C (OR 25.45), and West Haven grade III/IV (OR 2.23). Herbal medicine use and SBP showed non-significant associations with mortality. Multivariate logistic regression identified Child-Pugh class C13 (aOR 4.2, 95% CI 1.1-16.2; p = 0.043) and jaundice (aOR 3.5, 95% CI 1.1-11.0; p = 0.034) as independent predictors of mortality. West Haven grade was not significantly associated after adjustment (aOR 2.23, 95% CI 0.62-8.10; p = 0.220). These results are shown in Table 1.

**Table 1:** Bivariable and univariable logistic regression analysis of factors associated with mortality in hepatic encephalopathy

Variable	Bivariate OR (95% CI)	p-value	Multivariate aOR (95% CI)	p-value
Jaundice	6.29 (1.38–28.67)	0.017	4.2 (1.1 – 16.2)	0.043
Child-Pugh Class C	25.45 (3.17–204.26)	<0.001	3.5 (1.1 – 11.0)	0.034
West Haven Gr. III/IV	2.23 (0.76–6.60)	0.143	2.23 (0.62–8.10)	0.220
Herbal Medicine Use	1.84 (0.65–5.24)	0.250	1.8 (0.6 – 5.4)	0.30
Spontaneous Bacterial Peritonitis	1.70 (0.60–4.78)	0.320	1.5 (0.5 – 4.5)	0.45

A summary of patients’ demographics, clinical features, and outcomes is shown in Table 2.

**Table 2:** Summary of patients’ demographics, clinical features, and outcomes

Parameter	Value
Number of patients	69
Mean age (years)	49.8 ± 16.6
Male patients	49 (71.0%)
Female patients	20 (29.0%)
Cirrhosis prevalence	57 (82.6%)
Child-Pugh class C	43 (73.9%)
Asterixis present	41 (59.4%)
West Haven stage II	41 (59.4%)
Edematous-ascitic decompensation	52 (75.4%)
Jaundice	51 (73.9%)
Herbal medicine use	22 (31.9%)
Spontaneous bacterial peritonitis (SBP)	18 (26.1%)
Gastrointestinal bleeding	13 (18.8%)
Mortality	53 (76.8%)

## Discussion

General characteristics

HE is a severe complication of acute and chronic liver failure. It represents a pivotal turning point in the clinical management of cirrhotic patients. In our study, the hospital prevalence of HE was 2.8%, a finding consistent with other African series where the incidence of HE ranged from 6.5% to 13.5% [13,14,16]. A higher prevalence is reported in Western countries, varying between 30% and 45% among cirrhotic patients during follow-up [2]. This discrepancy may be explained by the hospital-based nature of our recruitment, as well as by the lack of systematic measurement of blood ammonia levels and screening tests for minimal HE in our setting.

The studied population was relatively young, with a mean age of 49 years, reflecting epidemiological data from the African continent, characterized by a generally younger population than in Western countries. A male predominance was observed, with a sex ratio of 2.45, a pattern frequently reported in African studies that may relate to higher exposure of men to hepatotoxins, alcohol, and later healthcare seeking [13,14,16-18].

Clinical and etiological features

One of the major findings in our study was the high frequency of asterixis, observed in 59.4% of patients. Asterixis, or “flapping tremor,” is a typical and nearly pathognomonic neuromuscular manifestation of HE, particularly useful in the clinical setting. It consists of an involuntary, arrhythmic flexion-extension movement of the wrists or fingers during sustained posture, reflecting a central disturbance in muscle tone regulation [5].

In resource-limited environments where access to investigations such as electroencephalography, neurocognitive scoring, or ammonia measurement is not routine, asterixis constitutes a crucial, accessible diagnostic sign. Its early recognition allows rapid suspicion of HE, especially among cirrhotic patients presenting with altered vigilance or behavioral changes.

For optimal monitoring of HE, clinicians should specify its pathophysiological mechanism (acute liver failure, porto-systemic shunts without liver disease, or cirrhosis with or without porto-systemic shunts), perform clinical staging using the West Haven classification, and identify precipitating factors [19].

All our patients presented with clinical HE. This may be explained by limited technical capabilities for detecting minimal HE. In such contexts, asterixis, which typically appears from stages I-II of the West Haven classification, a potentially reversible phase can serve as an early diagnostic indicator [19]. Early detection consequently allows initiation of treatment before progression to more severe stages (III-IV), which are often associated with high mortality.

Etiologically, cirrhosis was the main cause of HE in our cohort, identified in 82.6% of cases. This aligns with the literature, where cirrhosis is described as the primary cause of chronic HE, accounting for 80-90% of cases in Western series [2,11]. The high prevalence of cirrhosis in our population is largely explained by the endemicity of hepatitis B and C viruses in Senegal, as well as by the impact of hepatotoxins (notably herbal medicine, non-prescribed medications, and in some cases alcohol).

The severity of cirrhosis, as assessed by the Child-Pugh score, was marked in our series: 73.9% of patients were classified as Child-Pugh class C, the most advanced stage. At this level, hepatic regenerative capacity is overwhelmed, and ammonia detoxification mechanisms, including the urea cycle and astrocytic metabolism, are severely impaired, facilitating the onset of HE [5]. This high proportion of class C patients suggests late management of cirrhosis in our context, linked to diagnosis frequently made at the stage of complications (ascites, gastrointestinal bleeding, HE), but also to delayed healthcare access for socioeconomic, cultural, or geographic reasons.

Among identified precipitating factors, herbal medicine use accounted for a significant proportion (31.9%), ahead of SBP and gastrointestinal hemorrhages [20]. This observation merits particular attention as it highlights the impact of traditional practices in our context and underscores the need for patient education and monitoring regarding the use of non-conventional therapeutic products in cirrhotic patients [21].

Management, outcomes, and prognostic factors

Treatment of HE was primarily based on the combination of lactulose and rifaximin, consistent with international guidelines [22]. It has also been shown that controlling one or more precipitating factors, present in about half of cases, improves symptoms in approximately 90% of patients [23]. However, despite these treatments, clinical evolution was unfavorable in 76.8% of cases, with an elevated mortality rate of 76.8% and a mean time to death of 7.2 days after symptom onset. This mortality rate is comparable to some African series: 66.7% in Bamako, 63.2% in Madagascar, and 56% in Burundi in studies focusing on HE in chronic liver disease [13,14,24]. This rate is substantially higher than those reported in developed countries, where mortality varies between 20% and 40% depending on disease stage and treatment timeliness [22]. These findings likely reflect delayed management, limited technical resources, and severity of underlying hepatic diseases.

Multivariate analysis identified two factors independently associated with mortality among HE patients: a Child-Pugh score of class C13 (p = 0.043) and the presence of clinical jaundice (p = 0.034). These two factors are readily accessible in clinical practice, reinforcing their utility at the bedside for rapid risk stratification and management adaptation. Our results thus confirm the importance of simple yet robust prognostic tools for cirrhotic patients with HE in resource-limited settings.

Limitations of the study

This study has several limitations inherent to its retrospective design, including selection bias and incomplete medical records. Furthermore, the absence of systematic ammonia measurement or standardized neurocognitive follow-up may limit diagnostic accuracy in mild forms of HE. Another limitation is our relatively small sample size, which reflects the single-center nature of the study and may restrict the generalizability of our findings. Additionally, certain precipitating factors or comorbidities may have been underdocumented due to variability in record-keeping and the availability of diagnostic tools. Nevertheless, the study provides a realistic overview of care conditions in our context and serves as a useful foundation for future prospective research.

## Conclusions

HE remains a frequent and severe complication of cirrhosis in our context, characterized by high mortality. This study highlights the occurrence of HE in relatively young patients, predominantly at advanced stages of liver disease (Child-Pugh C), and often exposed to avoidable precipitating factors such as herbal medicine use.

The severity of outcomes, with nearly 77% mortality, underscores the importance of early diagnosis, prompt and appropriate management, and especially prevention strategies focusing on therapeutic patient education. Combating uncontrolled traditional practices, improving access to specialized care, and integrating standardized protocols in internal medicine and hepato-gastroenterology departments constitute key avenues to improve HE prognosis. Prospective multicenter studies are needed to refine understanding of HE in sub-Saharan Africa by incorporating more precise biological and neurocognitive data and to evaluate the impact of targeted intervention strategies in our healthcare settings.
